# Bletilla striata polysaccharide induces autophagy through PI3K/AKT signaling pathway to promote the survival of cross-boundary flap in rats

**DOI:** 10.3389/fphar.2025.1544932

**Published:** 2025-03-10

**Authors:** Qin Yue, Xinyi Zeng, Minlan Yang, Jinhao Chen, Lin Liu, Hui Liu

**Affiliations:** Yangtze University Health Science Center, Jingzhou, China

**Keywords:** Bletilla striata polysaccharide, PI3K/AKT, autophagy, cross-boundary flap, angiogenesis

## Abstract

**Introduction:**

Distal flap necrosis is a common problem in flap transplantation. Bletilla striata polysaccharide (BSP) is the main medicinal component of traditional Chinese medicine Bletilla striata. The purpose of this study was to investigate the mechanism of BSP promoting flap survival.

**Methods:**

The control group, BSP low, medium and high dose groups, BSP + autophagy inhibitor 3-methyladenine (3-MA) group were designed to establish a model of cross-boundary flap in rat back. After 7 days of postoperative administration, the samples were taken.

**Results:**

The optimal dose of BSP was determined to be 250 mg/kg/d according to the survival rate of flap, microvessel density, intra-arterial diameter, expression of vascular-related protein and pharmacological toxicity. By detecting the expression level of autophagy-related proteins, it was found that BSP could activate autophagy. After autophagy was blocked, the therapeutic effect of BSP was reversed. In addition, BSP activated the PI3K/AKT signaling pathway.

**Discussion:**

Studies have shown that BSP induces autophagy by activating PI3K/AKT signaling pathway, thereby promoting angiogenesis and improving survival rate of flap.

## 1 Introduction

As an important repair and reconstruction surgery, flap transplantation has been widely used in the medical field, which can help patients restore their appearance and have flexibility (Gu et al., 2024). However, due to operation, apoptosis, oxidative stress, ischemia-reperfusion injury and inflammatory response (Berry et al., 2024; Zhang D. P. et al., 2023; Dastagir et al., 2023), the distal function of flap is lost and ischemic necrosis occurs after oper ation (Bi et al., 2023). Therefore, promoting angiogenesis and ensuring distal blood supply are the key to improve the survival rate of flap (Su et al., 2024).

Autophagy is an important cellular physiological process for intracellular degradation and recycling of cellular components (Gumpper et al., 2019). It plays a key role in maintaining intracellular homeostasis, regulating cell growth and metabolism, and coping with environmental stress (Eskelinen and Saftig, 2009). Phosphoinositide 3-kinase (PI3K)/protein kinase B (AKT) signaling pathway is an important signal transduction pathway in cells, the activation of this signaling pathway can promote cell growth, proliferation and survival, and participate in the regulation of cell metabolism and stress response (Jiang et al., 2024). Recent researchs have shown that the activity of the PI3K/AKT signaling pathway induces autophagy, thereby promoting flap survival (Jiang et al., 2021; Chen T. X. et al., 2023). Autophagy can enhance angiogenesis (Lin et al., 2024), reduce inflammation, inhibit oxidative stress and pyroptosis (Wu et al., 2024a; Li et al., 2024). Therefore, inducing autophagy is an effective strategy to promote angiogenesis, reduce oxidative stress and improve the survival rate of flap.

Bletilla striata polysaccharide (BSP) is the main active ingredient of traditional Chinese medicine Bletilla striata, which is polymerized from α-mannose, β-mannose and β-glucose (Li et al., 2021). Studies have shown that BSP can inhibit the release of inflammatory mediators and the occurrence of inflammatory responses (Zhang L. L. et al., 2023). BSP has antioxidant activity, can scavenge free radicals and reduce the damage caused by oxidative stress (Li et al., 2020). BSP can also improve lipopolysaccharids-induced acute respiratory distress syndrome by inhibiting pyrodeath (Wu et al., 2024b). BSP can effectively treat bacterial infections (Zeng et al., 2023). In addition, BSP can reduce apoptosis and improve myocardial necrosis by activating PI3K/AKT signaling pathway (Wang J. Y. et al., 2022). BSP activates autophagy and alleviates pulmonary fibrosis by inhibiting TGF-β1/Smad signaling pathway (Wang K. T. et al., 2024). Previous studies have found that BSP can promote angiogenesis and improve the survival rate of flap (Yue et al., 2024). In view of this, it is worth exploring whether BSP can induce autophagy, promote angiogenesis and improve flap survival by activating PI3K/AKT signaling pathway.

## 2 Materials and methods

### 2.1 Reagents

BSP (purity ≥ 99%, batch number ZLSW211123-3, Xi ’an Zelang Biotechnology Co., Ltd.). 3-MA (MCE,United States). RIPA lysate, ECL protein luminescent reagent (Meilun Biotechnology Co., Ltd.). SDS-PAGE gel electrophoresis preparation kit, BCA protein quantitative kit (Shanghai Beyotime Biotechnology Co., Ltd.). Mouse anti-vascular endothelial growth factor (VEGF), glyceraldehyde-3-phosphate dehydrogenase (GAPDH), PI3K, vascular endothelial cadherin (CDH5), rabbit anti- endothelial nitric oxide synthase (eNOS), AKT, vacuolar protein sorting 34 (VPS34), matrix metalloproteinase 9 (MMP9), microtubule-associated protein light chain 3 (LC3) antibody, HRP goat anti-mouse secondary antibody, HRP goat anti-rabbit secondary antibody (Proteintech Group, Inc.).

### 2.2 Animals

The study protocol was approved by the Laboratory Animal Ethics Committee of the Yangtze University Health Science Center. A total of 30 clean grade (SPF) male Sprague Dawley rats, weighing (210 ∼ 230) g, were purchased from the Animal Center of China Three Gorges University, License No. : SCXK (Hubei) 20220012, and raised in a standardized animal house. The rats were randomly divided into control group, BSP-L group, BSP-M group, BSP-H group and BSP + 3-MA group, with 6 rats in each group. After the establishment of flap model, the BSP-L group, the BSP-M group and the BSP-H group were intraperitoneally injected with 125 mg/kg/d, 250 mg/kg/d and 500 mg/kg/d, respectively, The control group was given an equal dose of normal saline for 7 days. The BSP + 3-MA group was injected with 15 mg/kg/d 3MA, 30 min later, intraperitoneal injection of BSP.

On the 7th day after operation, the flap necrosis of rat in each group was observed. Through lead oxide angiography, hematoxylin and eosin (H&E) staining and Western blot analysis, the optimal medicinal concentration of BSP on the rat flap was screened first, and the related mechanism was discussed in combination with BSP + 3-MA group.

**FIGURE 1:**
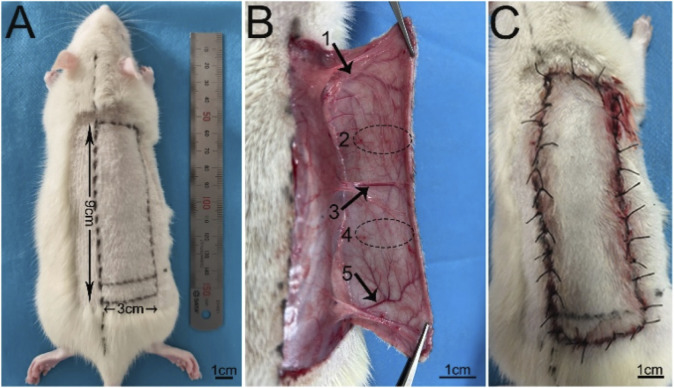
The design of the flap model **(A)** Design of 3 cm × 9 cm flap. **(B)** 1, 2, 3, 4 and 5 represent the thoracic dorsal vascular body (TDAV), choke II area, posterior intercostal vascular body (PIAV), choke I area, and iliolumbar vascular body (ILAV), respectively. **(C)** Flap suturing.

Before operation, rats were anesthetized by intraperitoneal injection of 1% pentobarbital sodium (40 mg/kg). A cross-boundary flap model with a width of 3 cm and a length of 9 cm rectangular frame (up to 0.5 cm below the scapula and down to 1 cm below the iliac crest) was designed on the right side of the rat back [22] (Figure 1A), which included thoracolumbar artery, posterior intercostal artery, iliolumbar artery and two choke areas (Figure 1B). The dorsal thoracic artery and posterior intercostal artery were ligate, and the iliolumbar artery was preserved. After the operation, the suture was sutured *in situ* with 4–0 lines, and the suture situ was disinfected with iodophor (Figure 1C). The modeling was completed by one person to reduce the errors. The rats were fed in single cages and free to eat.

### 2.3 The survival rate of flap was observed and counted

On the 7th day after operation, the hair, color, texture and other conditions of the flap were evaluated. There were scabs in the necrotic area, the color was brown or dark brown, the texture became hard, and the new hair was less. The digital camera was used to take photos, and the survival rate of flap was calculated by ImageJ software. The survival rate of flap = the survival area of flap/the total area of flap × 100% (Chen X. K. et al., 2023).

### 2.4 Gelatin lead oxide angiography

On the 7th day after operation, the rats were anesthetised and fixed, and 5% gelatin lead oxide was injected into one side of the common carotid artery with a No. 24 indenting catheter until the sclera and extremities of the rats were orange-red. The perfusion was stopped, and the rats were placed in a refrigerator at −20°C overnight, and the skin was removed after the contrast agent was solidified (Chen X. K. et al., 2023). X-ray imaging device was used to make a contrastive analysis of blood vessels.

### 2.5 H&E staining

The flap tissue in choke 1 area was taken in 1 cm × 0.5 cm, fixed with 4% paraformaldehyde, paraffin embedded, sliced and H&E staining. Three areas with relatively dense microvessels and the largest diameter in the arterial lumen were found under the low magnification (×100 ), and the number of microvessels was counted under the low magnification (×200 ), and the diameter of the arterial lumen was measured by ImageJ (Zhang Q. et al., 2023).

### 2.6 Western blot

The flap tissue in choke 1 area was taken in 30 mg and homogenized with 200 μL lysate. The protein concentration was determined and quantified. SDS-PAGA gel electrophoresis was performed, then wet-transferred to PVDF membrane, The membrane was blocked with 5% skimmed milk powder for 1h, and then washed with TBST, and incubated with primary antibodies VEGF (1:2,000), eNOS (1:1,000), PI3K (1:2,000), AKT (1:5,000) and GAPDH (1:10,000) at 4°C overnight, The membrane was washed with TBST for 3 × 5 min. HRP goat anti-mouse or goat anti-rabbit secondary antibody (1:5,000) was added and incubated at room temperature for 1 h. After washed with TBST for 3 × 10 min, the gel imaging system was used for development, and the experiment was repeated for three times. The gray value of protein bands was analyzed by ImageJ software (Zhang D. P. et al., 2023).

### 2.7 Bioinformatics analysis

Obtained the dataset (GSE35270) through the GEO website (https://www.ncbi.nlm.nih.gov/geo/). After logarithmic processing, the standardized expression level matrix of differentially expressed genes was screened. Performed differential analysis between the control group and the normal group samples using R language. The filtering criteria were: | log2(Fold Change) | > 1, P < 0.05. And further performed Gene Set Enrichment Analysis (GSEA).

### 2.8 Statistical analysis

All the data were statistically processed by Graph Pad Prism 8 software. The measurement data of normal distribution were expressed as mean ± standard deviation (‾x ± s). One-way analysis of variance was used among all groups, and p < 0.05 was considered statistically significant.

## 3 Result

### 3.1 Bioinformatics analysis

**FIGURE 2:**
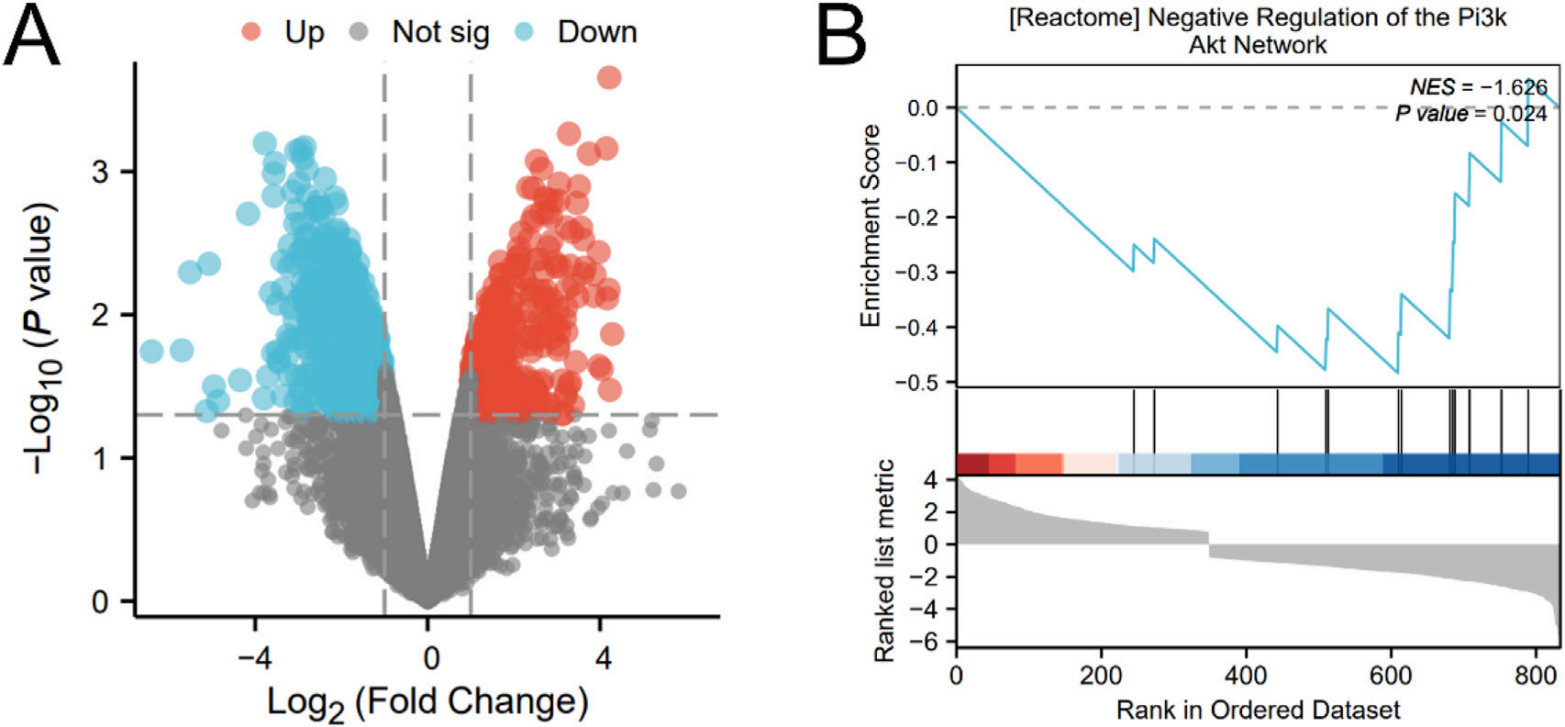
The result of bioinformatics analysis **(A)** Volcano plot of differentially expressed genes. **(B)** GSEA about PI3K/AKT signaling pathway.

In order to visually observe the distribution of upregulated and downregulated genes, a volcano plot of gene differences between samples was constructed (Figure 2A). Performed GSEA on differentially expressed genes and investigated the PI3K/AKT signaling pathway. The results showed that the PI3K/AKT signaling pathway was significantly enriched (Figure 2B). It showed that vasoconstriction can cause downregulation of the PI3K/AKT signaling pathway after modeling, thereby inhibiting autophagy.

### 3.2 BSP increases the survival rate of flap

**FIGURE 3:**
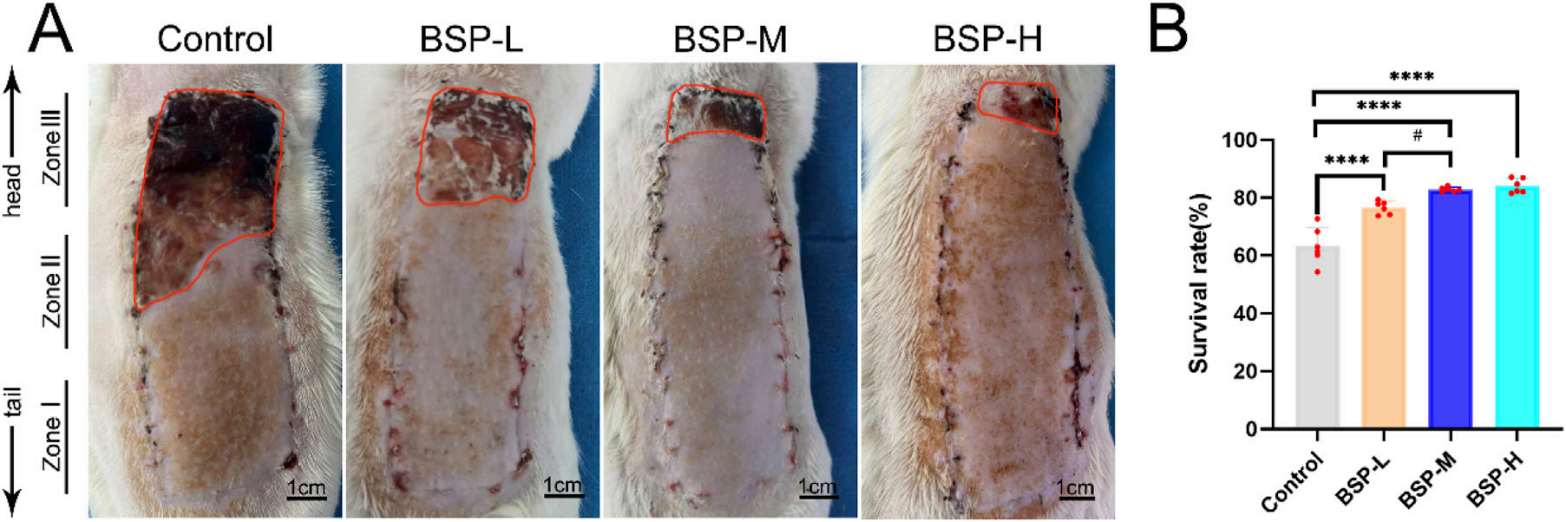
BSP increases the survival rate of flap **(A)** The flap survival of rats in each group on the 7th day after operation. **(B)** Statistical chart of survival rate of flap in each group. Compared with the control group, ****p < 0.0001, compared with the BSP-M group, ^#^p < 0.05, n = 6.

On the 7th day after operation, there was no necrosis in the zone I of flaps in each group, and the texture was soft and elastic with no scar. Compared with BSP-L, BSP-M and BSP-H, the necrosis of flap in the control group reached zone II, and showed brown scars with hard and inelastic texture (Figure 3A). The results showed that the survival rate of flap in control group (63.31% ± 5.86%) was significantly different from that in BSP-L (76.56% ± 2.08%), BSP-M (82.82% ± 0.72%) and BSP-H (84.13% ± 2.21%) groups (p < 0.0001). In addition, the survival rate of flaps in BSP-M and BSP-H groups was higher than that in BSP-L group (p < 0.05), but there was no statistical difference in the survival rate of flaps between BSP-M and BSP-H groups (Figure 3B). It can be seen that BSP can significantly increase the survival area of flaps, and there is a certain relationship with the dose.

### 3.3 BSP has a positive effect on angiogenesis

**FIGURE 4:**
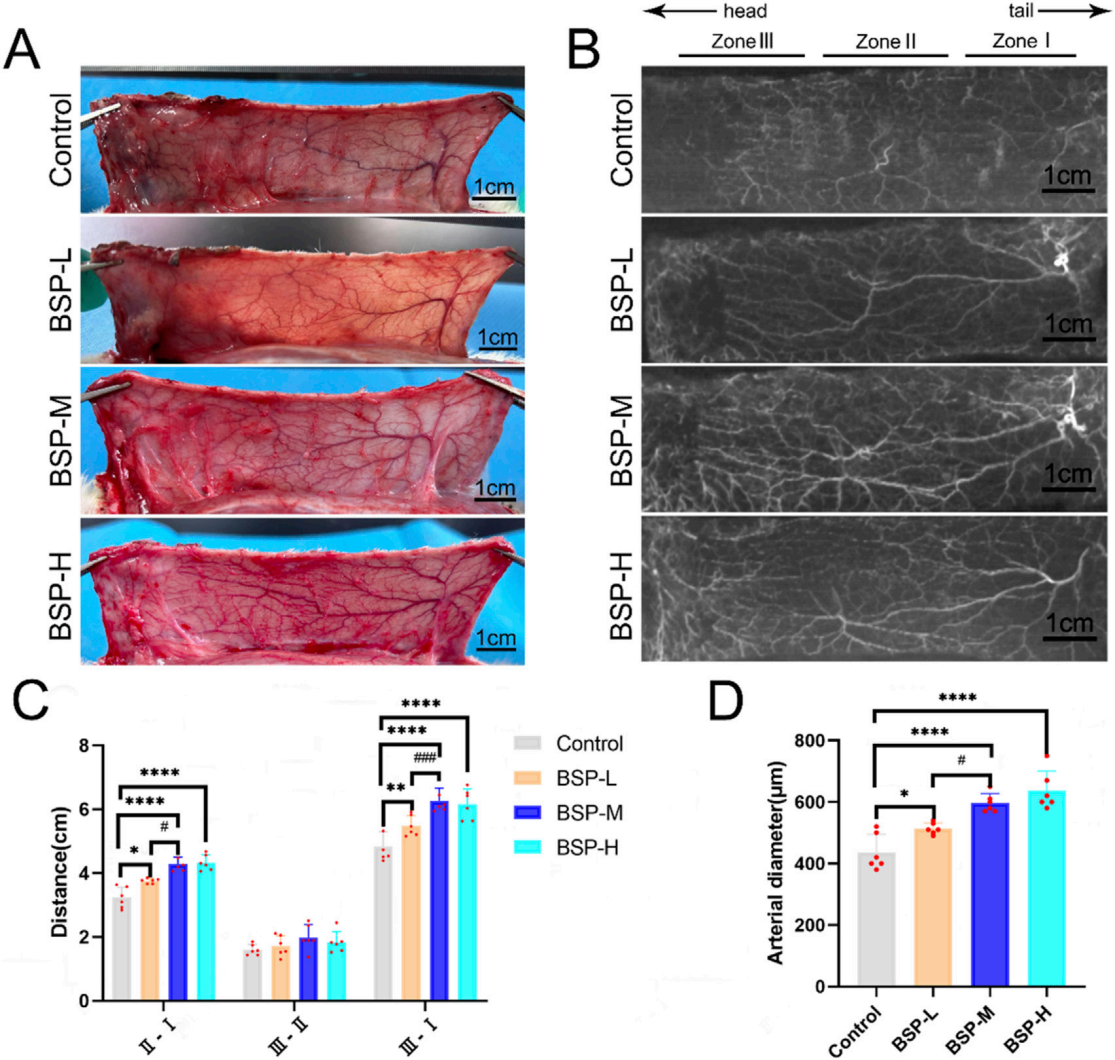
The effect of BSP on blood vessels **(A)** Vascular morphology of the flaps in each group on the 7th day after operation. **(B)** Gelatin lead gelatin oxide angiography of flap in each group on the 7th day after operation. **(C)** Statistical chart of vascular distance of the flaps in each group. **(D)** Statistical chart of diameter of the iliolumbar artery in each group. Compared with the control group, *p < 0.05, **p < 0.01, ****p < 0.0001, compared with the BSP-M group, ^#^p < 0.05, ^###^p < 0.001, n = 6.

On the 7th day after operation, the observation of blood vessels in flap and the results of gelatin lead oxide angiography showed that compared with the control group, the blood vessels in the BSP-L, BSP-M and BSP-H groups were rich and the vascular structure was clear (Figures 4A, B). The distance between the PIAV and the ILAV was measured (II - I), and it was found that the control group (3.23 cm ± 0.30 cm) was significantly different from that in BSP-L (3.76 cm ± 0.07 cm), BSP-M (4.28 cm ± 0.20 cm) and BSP-H (4.33 cm ± 0.22 cm) groups (p < 0.05, Figure 4C). However, there was no significant difference in the distance between the TDAV and the PIAV (III - II) in control group (1.60 cm ± 0.16 cm) and BSP-L (1.72 cm ± 0.30 cm), BSP-M (1.99 cm ± 0.37 cm) and BSP-H (1.84 cm ± 0.30 cm) groups. The distance between the TDAV and the ILAV (III - I) in control group (4.83 cm ± 0.42 cm) was smaller than that in BSP-L (5.48 cm ± 0.30 cm), BSP-M (6.27 cm ± 0.36 cm) and BSP-H (6.16 cm ± 0.43 cm) groups. There were significant differences (p < 0.05, Figure 4C). BSP can lengthen the vascular extension to the distal end of flap, mainly by lengthening the distance between the PIAV and the ILAV, which may be related to the enlargement of the vascular inner diameter and the increase of microvascular density in choke I area.

The diameter of the iliolumbar artery in the control group, BSP-L, BSP-M and BSP-H groups were (436.66 ± 53.44) μm, (513.33 ± 17.00) μm, (596.67 ± 28.09) μm, (636.67 ± 57.93) μm, respectively. The diameter of the iliolumbar artery in the BSP-L, BSP-M and BSP-H groups was much higher than that in control group, and the difference was significant (p < 0.0001, Figure 4D).

**FIGURE 5:**
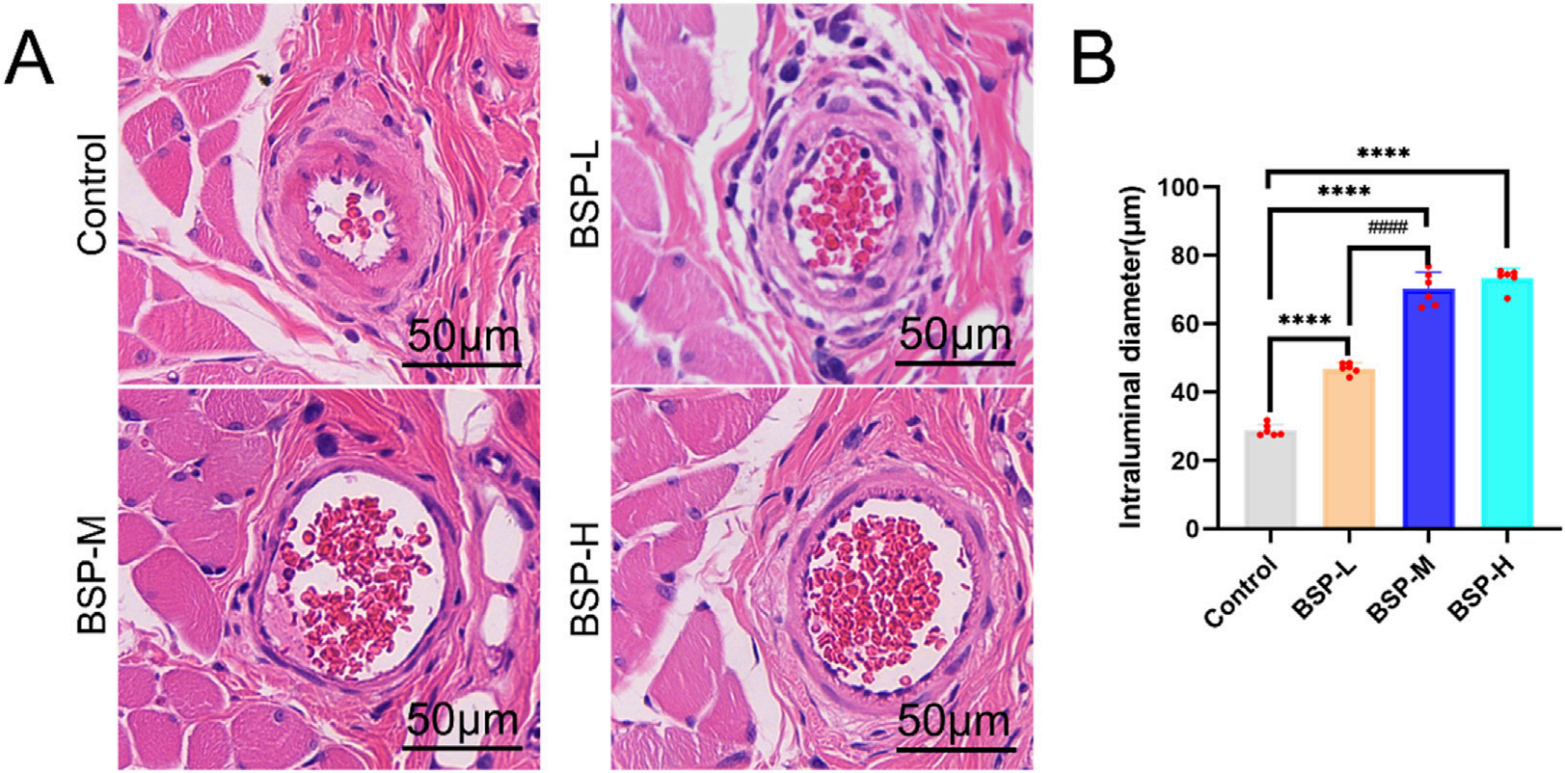
The effect of BSP on vascular inner diameter **(A)** H&E staining showed the vascular inner diameter. **(B)** Statistical chart of vascular inner diameter in each group. Compared with the control group, ****p < 0.0001, compared with the BSP-M group, ^####^p < 0.0001, n = 6.

The vascular inner diameter of choke I area was measured by H&E staining (Figure 5A). The vascular inner diameter of control group, BSP-L, BSP-M and BSP-H groups were (28.86 ± 1.57) μm, (46.95 ± 1.42) μm, (70.20 ± 4.46) μm, and (73.35 ± 2.68) μm, respectively. The vascular inner diameter of choke I area in BSP-L, BSP-M and BSP-H groups was significantly higher than that in control group (p < 0.0001, Figure 5B), and the vascular inner diameter in BSP-M group was higher than that in BSP-L group (p < 0.05, Figure 5B). There was no statistical difference between BSP-M group and BSP-H group.

**FIGURE 6:**
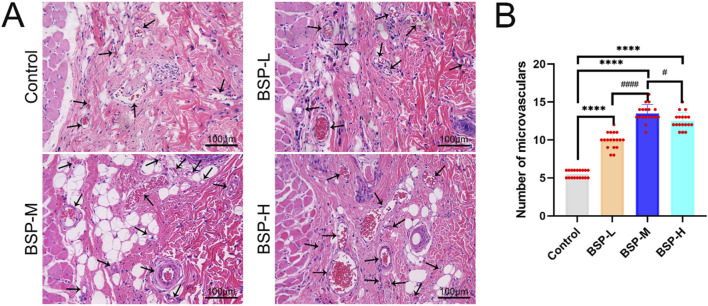
The effect of BSP on the number of microvessels **(A)** H&E staining showed the number of microvessels. **(B)** Statistical chart of the number of microvessels in each group. Compared with the control group, ****p < 0.0001, compared with the BSP-M group, ^#^p < 0.05, ^####^p < 0.0001, n = 6.

H&E staining showed the number of microvessels in choke I area (Figure 6A), found that BSP-L (9.94 ± 1.03), BSP-M (13.50 ± 1.12), and BSP-H (12.50 ± 1.07) groups was much higher than that the control group (5.50 ± 0.50), and there was a significant difference (p < 0.0001, Figure 6B). In the experimental group, the number of microvessels in BSP-M group was higher than that in BSP-L group and BSP-H group (p < 0.05, Figure 6B).

**FIGURE 7:**
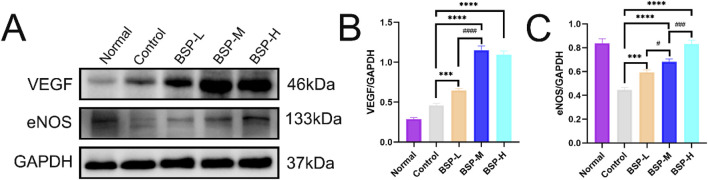
BSP upregulated the expression of VEGF and eNOS protein **(A)** Western blot was used to detect the expression of VEGF and eNOS protein. **(B, C)** Statistical chart of protein quantitative analysis. Compared with the control group, ***p < 0.001, ****p < 0.0001, compared with the BSP-M group, ^#^p < 0.05, ^###^p < 0.001, ^####^p < 0.0001.

Western blot detected the expression levels of VEGF and eNOS in the flaps of the control group, BSP-L, BSP-M and BSP-H groups (Figure 7A). The expression levels of VEGF and eNOS in the BSP group were significantly higher than those in control group, and the expression level of VEGF in BSP-M group was higher than that in BSP-L group, while the expression level of eNOS was positively correlated with the concentration of BSP (p < 0.05, Figures 7B, C).

In summary, BSP has a positive effect on angiogenesis of cross-boundary flap in rat. Overall considering the statistical differences and pharmacological toxicity of the two groups. We finally determined that the optimal dose of BSP was 250 mg/kg/d.

### 3.4 BSP enhances autophagy of flap

**FIGURE 8:**
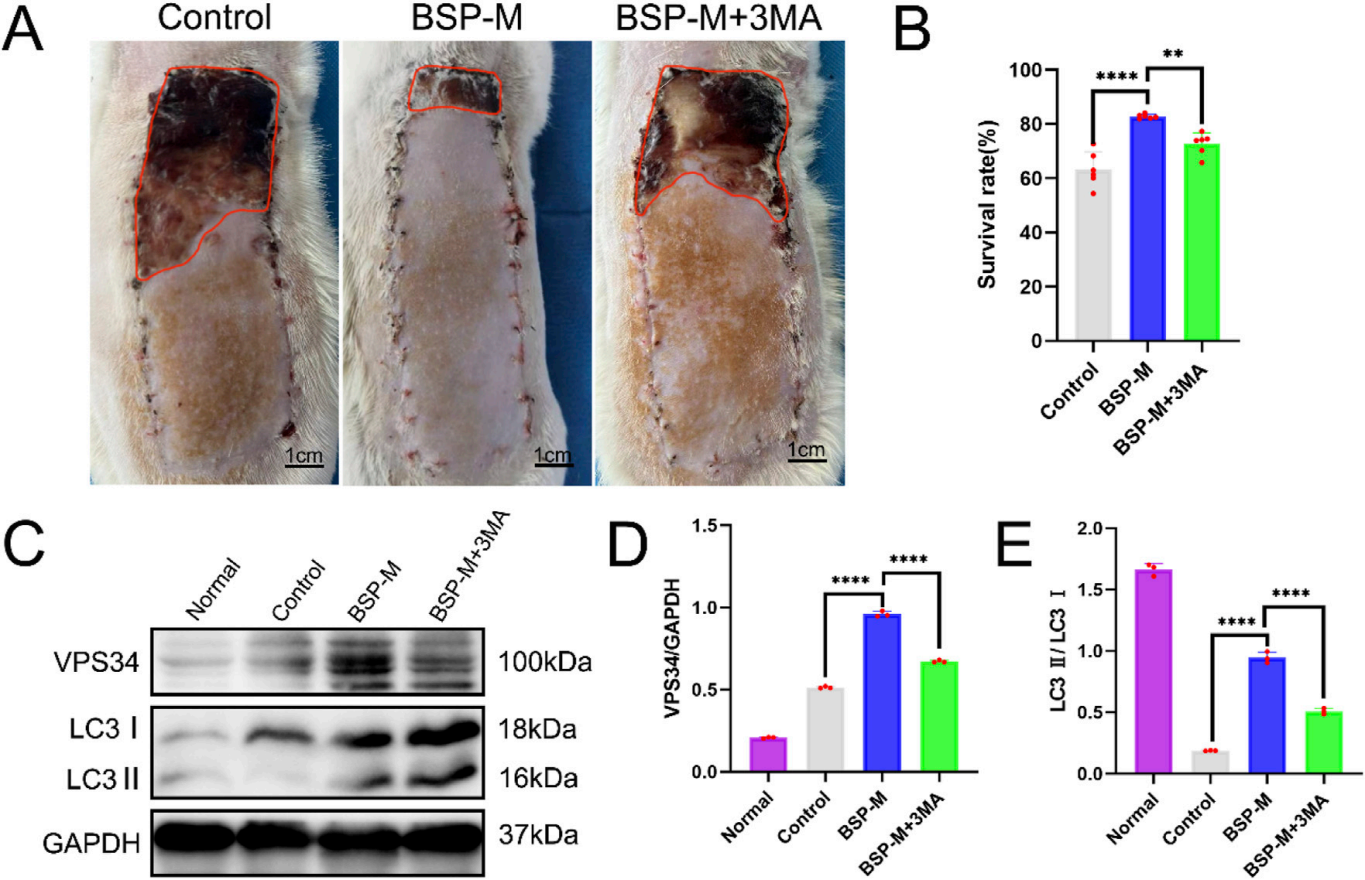
BSP enhances autophagy of flap **(A)** The flap survival of rats in each group on the 7th day after operation. **(B)** Statistical chart of survival rate of flap in each group. **(C)** Western blot was used to detect the expression of VPS34 and LC3 protein. **(D, E)** Statistical chart of protein quantitative analysis. Compared with BSP-M, **p < 0.01, ****p < 0.0001, n = 6.

In order to further study whether the positive effect of BSP on the flap is related to autophagy, the autophagy inhibitor 3-MA was used. On the 7th day after operation, there was necrosis occurred in the zone III of flap in the BSP-M + 3-MA group, showing brown hard scab, necrosis occurred in the distal end of flap in the zone II, no necrosis occurred in the zone I, and the skin was soft (Figure 8A). The results showed that the survival rate of flaps in BSP-M + 3-MA group was significantly lower than that in BSP-M group (72.69% ± 3.73% and 82.82% ± 0.72%, respectively, p < 0.01, Figure 8B). In addition, Western blot detected the expression levels of key autophagy proteins VPS34 and LC3 in the flaps of the control group, BSP-M group and BSP-M + 3-MA group (Figure 8C), and the expression levels of VPS34 and LC3 in the BSP-M group were higher than those in the control group and BSP-M + 3-MA group (p < 0.05, Figures 8D, E). The above results indicated that BSP enhanced autophagy of flap.

### 3.5 The inhibition of autophagy reverse the effect of BSP on blood vessels

**FIGURE 9:**
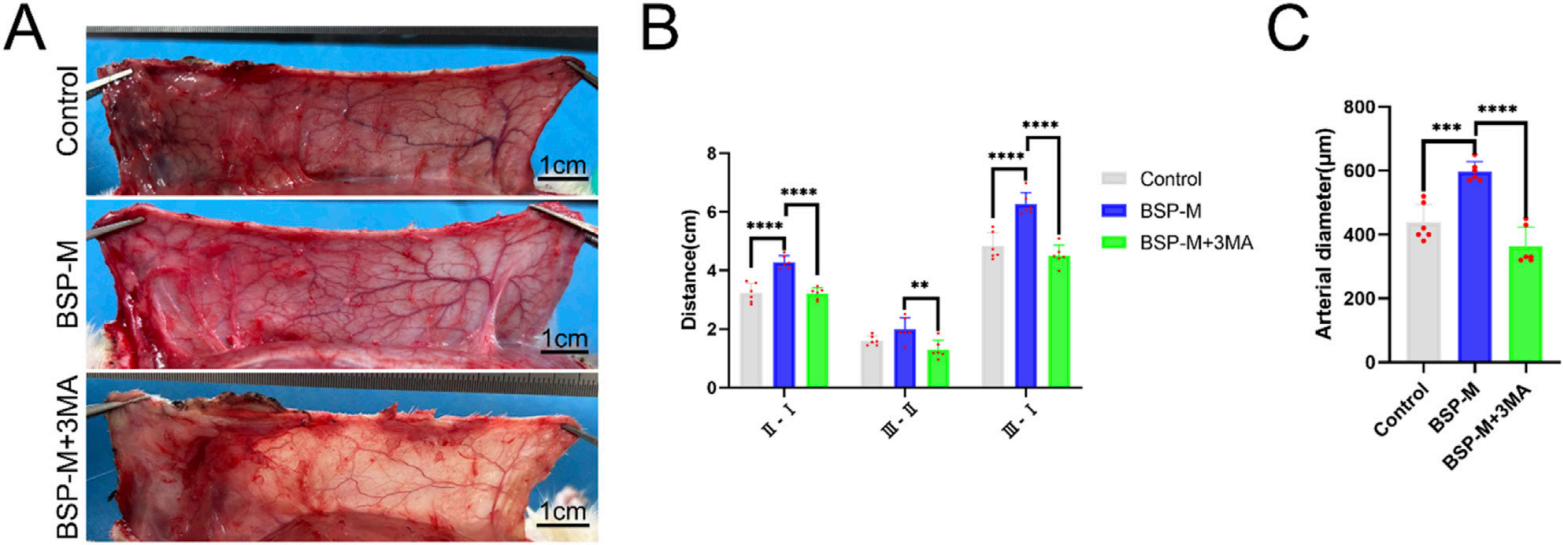
The inhibition of autophagy reverse the effect of BSP on blood vessels **(A)** Vascular morphology of the flaps in each group on the 7th day after operation. **(B)** Statistical chart of vascular distance of the flaps in each group. **(C)** Statistical chart of diameter of the iliolumbar artery in each group. Compared with BSP-M group, **p < 0.01, ***p < 0.001, ****p < 0.0001, n = 6.

**FIGURE 10:**
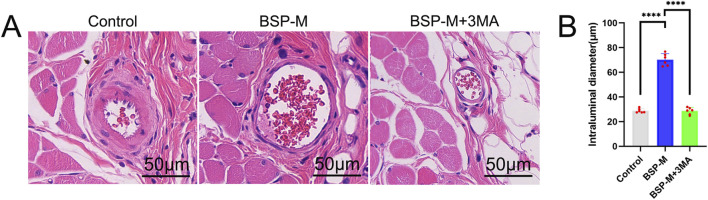
The inhibition of autophagy reverse the effect of BSP on vascular inner diameter **(A)** H&E staining showed the vascular inner diameter. **(B)** Statistical chart of vascular inner diameter in each group. Compared with BSP-M group, ****p < 0.0001, n = 6.

**FIGURE 11:**
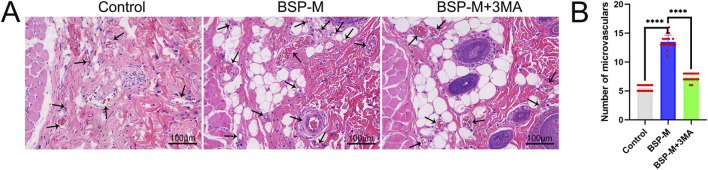
The inhibition of autophagy reverse the effect of BSP on the number of microvessels **(A)** H&E staining showed the number of microvessels. **(B)** Statistical chart of the number of microvessels in each group. Compared with BSP-M group, ****p < 0.0001, n = 6.

**FIGURE 12:**
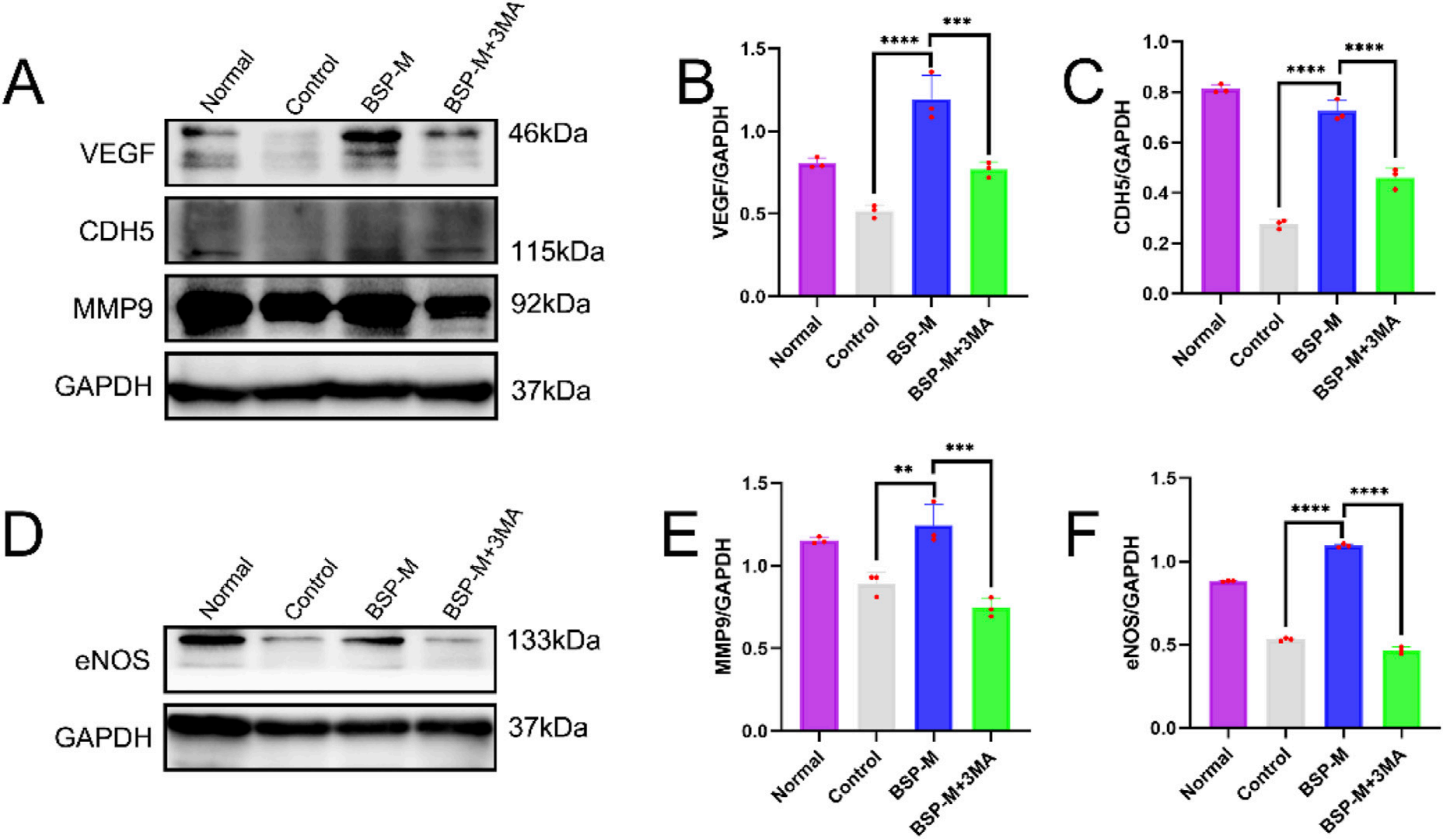
The inhibition of autophagy reverse the effect of BSP on blood vessels **(A, B)** Western blot was used to detecte the expression of VEGF,CDH5,MMP9 and eNOS protein. **(C–F)** Statistical chart of protein quantitative analysis. Compared with BSP-M group, **p < 0.01, ***p < 0.001, ****p < 0.0001, n = 6.

On the 7th day after operation, the observation of blood vessels in flap showed that the blood vessels in the BSP-M + 3-MA group were sparse and structurally disordered (Figure 9A). In the BSP-M + 3-MA group, II - I (3.22 cm ± 0.18 cm and 4.28 cm ± 0.20 cm, respectively, p < 0.0001), III - II (1.30 cm ± 0.29 cm and 1.99 cm ± 0.37 cm, respectively, p < 0.01), III - I (4.52 cm ± 0.33 cm and 6.27 cm ± 0.36 cm, respectively, p < 0.0001) were shorter than those in the BSP-M group (Figure 9B). It can be seen that 3-MA reversed the effect of BSP in promoting the distal extension of blood vessels in flap. The diameter of iliolumbar artery in BSP-M + 3-MA group (363.33 μm ± 54.67 μm) was much smaller than that in BSP-M group (596.67 μm ± 28.09 μm, p < 0.0001, Figure 9C). The inner diameter of blood vessels in choke I area (28.66 μm ± 2.57 μm) was significantly smaller than that in BSP-M group (70.20 μm ± 4.46 μm, p < 0.0001, Figures 10A, B), indicating that 3-MA inhibited vasodilation of flap. The number of microvessels in the choke I area of BSP-M + 3-MA group was less than that of BSP-M group (7.33 ± 0.67 and 13.50 ± 1.12, respectively, p < 0.0001, Figures 11A, B), indicating that 3-MA decreased the microvessel density in choke I area of flap. In addition, the results of Western blot showed that the expression levels of vascular-related proteins VEGF, CDH5, MMP9 and oxidative stress-related proteins eNOS in the BSP-M + 3-MA group were significantly lower than those in the BSP-M group (p < 0.001, Figures 12A–F). These results indicated that the inhibition of autophagy by 3-MA reverses the positive effect of BSP on blood vessels.

### 3.6 BSP induces autophagy by regulating PI3K/AKT signaling pathway

**FIGURE 13:**
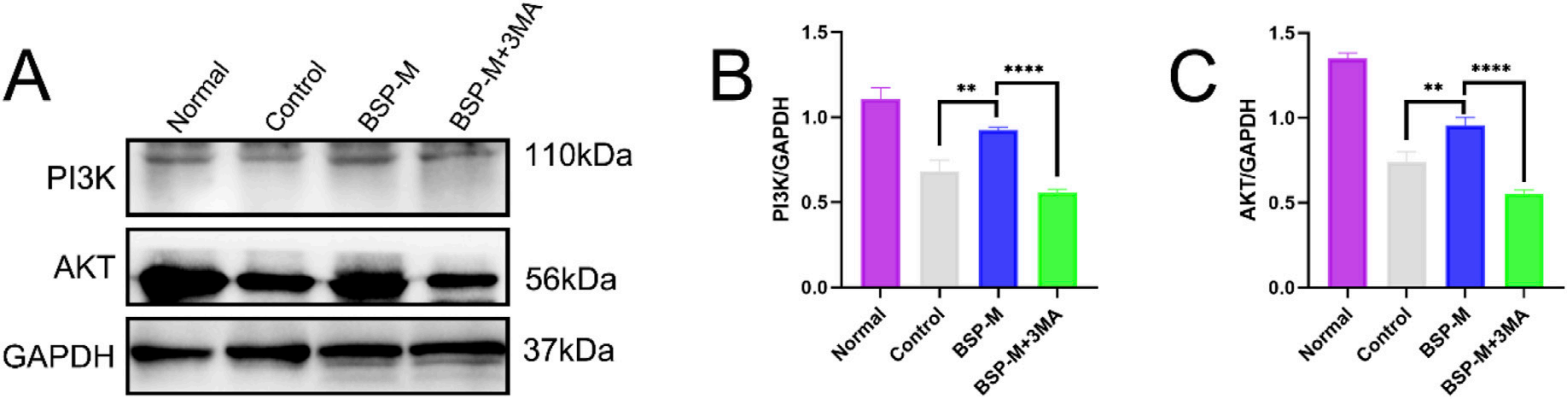
BSP regulates autophagy through PI3K/AKT pathway *in vitro* **(A)** Western blot was used to detect the expression of PI3K and AKT protein. **(B, C)** Statistical chart of protein quantitative analysis. Compared with BSP-M group, **p < 0.01, ****p < 0.0001.

Jianying Wang et al. discovered the effect of BSP on the PI3K/AKT signaling pathway (Wang L. et al., 2022). 3-MA is a selective autophagy inhibitor of the PI3K/AKT signaling pathway. Western blot detected and analyzed the expression of proteins related to PI3K/AKT signaling pathway in the flap (Figure 13A). Compared with the BSP-M group, the expression levels of PI3K and AKT in the BSP-M + 3-MA group were significantly reduced (p < 0.0001, Figures 13B, C). BSP can activate the PI3K/AKT signaling pathway to induce autophagy.

## 4 Discussion

The flap transplantation has been widely used in tissue repair under various conditions such as trauma, burn and tumor resection (Hsu et al., 2024; Liu et al., 2024). However, it is often difficult to heal wound in the donor site due to postoperative infection, insufficient blood supply and other reasons, resulting in flap necrosis (Rao et al., 2024; Eyuboglu et al., 2024). BSP is a natural, non-toxic and economical polysaccharide compound extracted from the root of Bletilla striata. It has pharmacological effects such as anti-inflammatory, anti-oxidation and promoting wound healing (Li et al., 2021; He et al., 2024; Jakfar et al., 2022). Previous studies have confirmed that BSP can promote flap survival [18], but there is no report on the mechanism of action of BSP on flap and autophagy, let alone the optimal medicinal concentration. In this study, the survival rate of flap and the expression level of vascular-related factors were significantly improved after BSP treatment, and were related to the drug dose. Overall considering the statistical differences and pharmacological toxicity of the two groups. The optimal medicinal concentration was 250 mg/kg/d.

The flap survival is based on continuous blood supply, and the angiogenesis is the key to the reconstruction of blood circulation of flap [6]. Angiogenesis is related to a variety of factors, including the regulation of oxidative stress (Huang and Nan, 2019) and a variety of endothelial factors (Escobar et al., 2004). eNOS is an antioxidant enzyme that plays an important role in reducing oxidative stress and increasing vasodilation (Lee and Im, 2021). VEGF is an important angiogenic factor, which increases vascular permeability and promotes angiogenic activity by promoting endothelial cell mitosis (Florek et al., 2024). Gelatin lead oxide angiography showed that the blood vessels of flaps in the BSP group extended longer to the distal end, and the arterial diameter became thicker. In addition, H&E staining showed that the microvessels of flaps in the BSP group increased significantly, and the inner diameter of the blood vessels also expanded significantly. Western blot further confirmed that BSP promoted the upregulation of expression levels of VEGF and eNOS. These findings indicate that BSP promotes angiogenesis and reduces oxidative stress by up-regulating VEGF and eNOS to improve growth of flap. Yan et al. (2018) confirmed that BSP could upregulate the expression of VEGF and promote angiogenesis through the perimenopausal rat model, which is consistent with our results.

**FIGURE 14:**
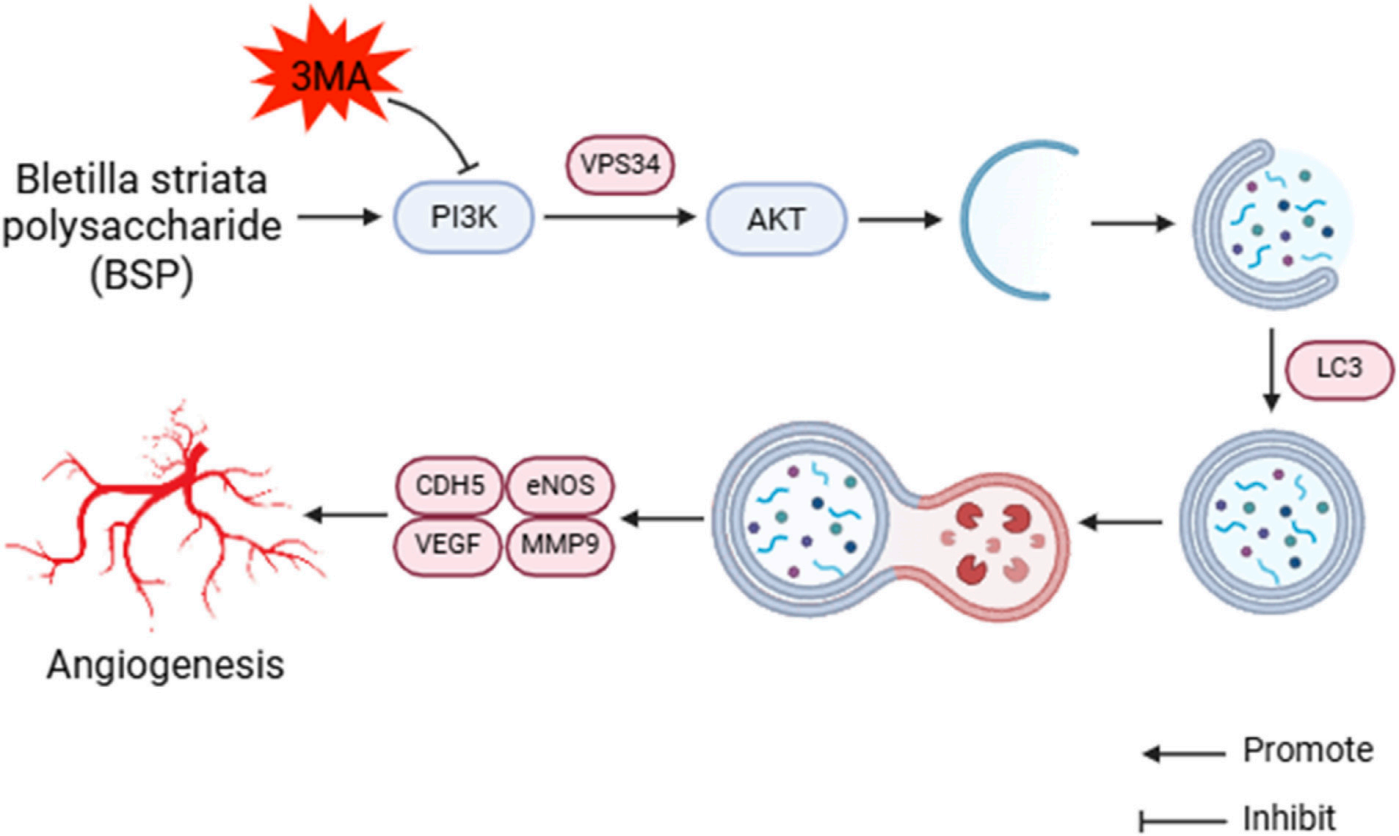
BSP induces autophagy to promote angiogenesis and inhibite oxidative stress by PI3K/AKT signaling pathway.

Autophagy is a process of cell self-degradation. It maintains the stability of the intracellular environment by encapsulating intracellular harmful or aging components in vesicles and transporting them to lysosomes for degradation. It plays an important role in multiple biological processes such as cell survival, metabolic regulation and immune response (Gao et al., 2022; Liu S. Z. et al., 2023). The PI3K/AKT signaling pathway is an important autophagy pathway (Liu et al., 2020). As the only member of class III PI3K, VPS34 plays an important role in regulating autophagy and macrophage phagocytosis (Liu Y. et al., 2023). LC3 is located in the middle reaches of the PI3K/AKT signaling pathway and is a key protein of autophagy. LC3 precursor molecules are cleaved to form cytoplasmic form LC3 I and further activated to form membrane-bound form LC3 II, which is involved in the formation and degradation of autophagosomes. LC3 II/I can be used as an evaluation indicator of autophagy level (Tanida, 2011; Tanida et al., 2008). CDH5 is an intercellular adhesion molecule involved in maintaining the integrity of endothelial cell and vascular permeability (Sauteur et al., 2014). MMP9 is an important matrix metalloproteinase, which plays a key role in the process of tumor invasion and metastasis, inflammation response and so on (Deng et al., 2022; Lin et al., 2022). CDH5, eNOS, VEGF and MMP9 are located downstream of the PI3K/AKT signaling pathway. Bioinformatics analysis showed that the PI3K/AKT pathway was downregulated after modeling, downstream gene expression was enriched, and autophagy was inhibited. 3-MA is a commonly used inhibitor of autophagy, which blocks the initiation of autophagy by inhibiting the activity of Class III PI3K enzyme in the PI3K signaling pathway (Petiot et al., 2000). We conjectured that BSP upregulates the expression of VPS34 and induces autophagy through activating PI3K/AKT signaling pathway, thereby up-regulating the expression of LC3, promoting the formation and degradation of autophagosomes, thereby enhancing the expression and secretion of VEGF, MMP9, CDH5 and eNOS, thereby promoting angiogenesis and dilatation, improving vascular permeability, reducing oxidative stress (Wang S. Q. et al., 2024; Elseweidy et al., 2023; Hung et al., 2016; Wang J. Y. et al., 2022; Zhang L. L. et al., 2023) and increasing the survival rate of flap (Figure 14).

This study found that the expression levels of VPS34 and LC3 II/I in the BSP-M group were higher than those in the control group, indicating that BSP increased the autophagy of flap. Moreover, the expression levels of VPS34 and LC3 II/I in the BSP-M + 3-MA group decreased after treatment with 3-MA, indicating that the combination of 3-MA and BSP could inhibit autophagy. In this study, it was found in Figures 5, 6 that 3-MA reversed the increase of survival rate of flap, microvessel density and the expansion of vascular inner diameter promoted by BSP, and even reversed the results of the expression levels of VEGF, MMP9, CDH5 and eNOS upregulated by BSP. It can be seen that BSP promotes angiogenesis of flap by activating autophagy. Figure 13 shows that BSP can activate the PI3K/AKT signaling pathway. Thus, these results confirm our conjecture that BSP can induce autophagy by activating PI3K/AKT signaling pathway, thereby promoting angiogenesis, inhibiting oxidative stress and improving the survival rate of flap.

There are still some limitations in this study. For example, this study only involved experiments of rat and did not conduct *in vitro* experiments. In addition, no positive control was set in this study. Nevertheless, this study provides strong evidence to support the benefits and mechanisms of BSP on flap survival. It laid the foundation for more effective clinical application of BSP.

## 5 Conclusion

In this study, it was confirmed that BSP induced autophagy by activating PI3K/AKT signaling pathway, thereby reducing oxidative stress, promoting angiogenesis and improving flap survival.

## Data Availability

The raw data supporting the conclusions of this article will be made available by the authors, without undue reservation.
