# Supplementary figures and images for: Bletilla striata polysaccharide induces autophagy through PI3K/AKT signaling pathway to promote the survival of cross-boundary flap in rats
Source: Front Pharmacol. 2025 Mar 10;16:1544932. doi: 10.3389/fphar.2025.1544932 (PMC11931138; doi:10.3389/fphar.2025.1544932)

Supplementary Material

# Original western blot for three repeats

**
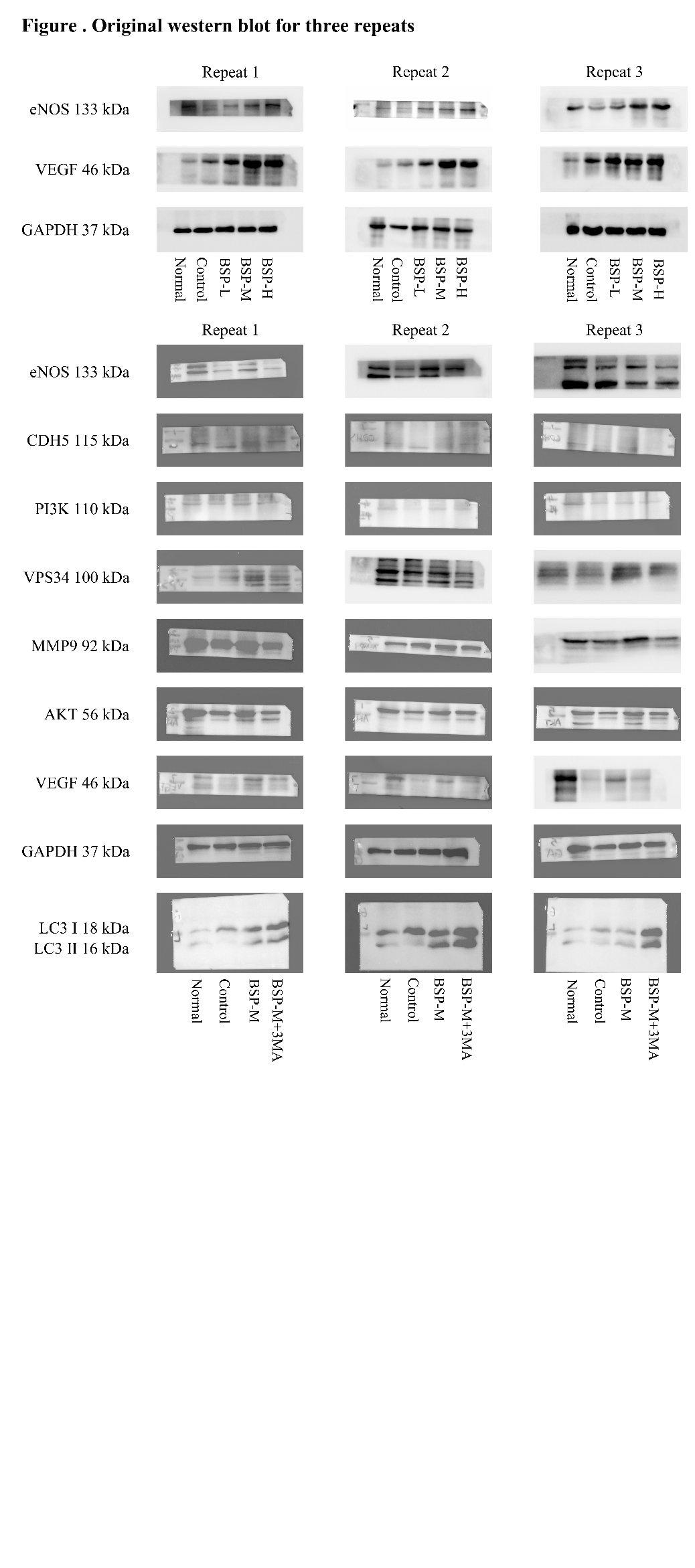
**

Supplement: Supplementary file 1 [file DataSheet1.docx]
